# Structural and functional differences in auto-antibody positive compared to auto-antibody negative hypothyroid patients with chronic thyroiditis

**DOI:** 10.1038/s41598-023-42765-z

**Published:** 2023-09-20

**Authors:** Yuichiro Iwamoto, Tomohiko Kimura, Takashi Itoh, Shigehito Mori, Taku Sasaki, Toshitomo Sugisaki, Erina Nakao, Mana Ohnishi, Takashi Kusano, Haruka Takenouchi, Hideyuki Iwamoto, Junpei Sanada, Yoshiro Fushimi, Yukino Katakura, Fuminori Tatsumi, Masashi Shimoda, Shuhei Nakanishi, Tomoatsu Mune, Kohei Kaku, Hideaki Kaneto

**Affiliations:** Division of Diabetes, Metabolism and Endocrinology, Kawasaki Medical School, 577 Matsushima, Kurashiki, 701-0192 Japan

**Keywords:** Endocrinology, Endocrine system and metabolic diseases, Thyroid diseases

## Abstract

Most primary hypothyroidism in adults is caused by chronic thyroiditis. Autoantibodies such as anti-thyroglobulin antibody (TgAb) and anti-thyroid peroxidase antibody (TPOAb) are involved in the pathogenesis of chronic thyroiditis. On the other hand, the clinical features of antibody-negative hypothyroidism are not clear. In this study, we aimed to determine the prevalence of thyroid-related autoantibodies in patients with primary hypothyroidism and to evaluate the differences in thyroid structure between antibody-positive and antibody-negative hypothyroidism. Among 804 patients who attended Kawasaki Medical School Hospital for thyroid hormone abnormalities or thyroid gland enlargement between January 1, 2010 and December 31, 2021, 237 patients with primary hypothyroidism who underwent thyroid antibody measurement and thyroid ultrasound examination were included. Participants were divided into groups according to antibody positivity/negativity, and differences in antibody positivity and thyroid structure were evaluated. In this study, 34.6% of patients had antibody-negative hypothyroidism. The positive rate of each antibody was 62.0% for TgAb and 49.4% for TPOAb. The participants with antibody-positive hypothyroidism had significantly larger thyroid gland on thyroid ultrasound examination (p < 0.05). Thyroid-stimulating hormone was significantly higher in participants with antibody-positive compared to antibody-negative hypothyroidism. The present study reveals a positive rate of thyroid-related autoantibodies in patients with hypothyroidism and the differences in thyroid structure between patients with and without antibodies. This study clearly show that the prevalence of antibody-negative chronic thyroiditis is quite high among hypothyroid patients, although this point needs confirmation by further investigations. The data in this study would be useful for the treatment of antibody-negative hypothyroid patients.

## Introduction

Primary hypothyroidism in adults is most often caused by chronic thyroiditis^1^. The diagnosis of chronic thyroiditis can be made by measuring anti-thyroglobulin antibody (TgAb) and/or anti-thyroid peroxidase antibody (TPOAb)^2^. Although it is clinically difficult to diagnose chronic thyroiditis when TgAb and TPOAb are negative, the improved accuracy of thyroid ultrasonography has improved the diagnostic yield of chronic thyroiditis by assessing the internal roughness of the thyroid gland, blood flow velocity and smoothness of the thyroid limbus^3^. Thus, primary hypothyroidism with negative TgAb and TPOAb may be treated in the same way as chronic thyroiditis. On the other hand, few studies have evaluated how thyroid ultrasound findings are obtained in patients with antibody-negative primary hypothyroidism and how these findings correlate with actual thyroid hormone levels and autoantibody levels. This study was designed to determine the prevalence of thyroid-related autoantibodies in patients with hypothyroidism and to evaluate structural differences in the thyroid gland between the presence and absence of thyroid-related autoantibodies.

## Materials and methods

### Study population and patient preparation

**Figure 1:**
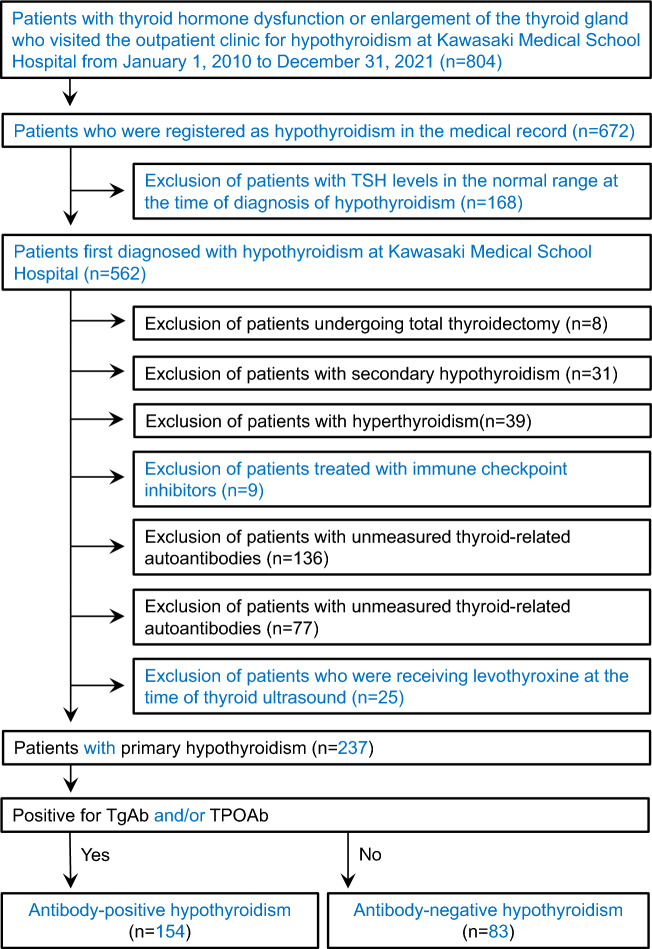
Flow chart regarding the participants in this study.

The study included 804 patients who visited the outpatient department of endocrinology at Kawasaki Medical School Hospital between January 1, 2010, and December 31, 2021, for abnormal thyroid-stimulating hormone (TSH) or free triiodothyronine (FT3) or free thyroxine (FT4), or for thyroid gland enlargement. The Institutional Review Board of Kawasaki Medical School (No. 5668–00) approved the study protocol, including opt-out informed consent. This study was conducted by the principles of the Declaration of Helsinki. The flow of study participants in this study is shown in Fig. 1. Among the first 804 recruited, 672 patients with primary hypothyroidism and disease registration in the electronic medical record were selected. The 168 patients who had normal TSH levels or were already taking levothyroxine at the time of disease registration were excluded from the study. Of the 562 patients diagnosed with subclinical or overt primary hypothyroidism at our institution, patients under 20 years of age with a history of Graves' disease, post-total thyroidectomy, and secondary hypothyroidism were excluded from the analysis (78 patients). Nine patients who received immune checkpoint inhibitors during the observation period were also excluded from the study. Patients with unmeasured TgAb and TPOAb (136 patients) and those without thyroid ultrasound (77 patients) were excluded from the analysis. We also excluded from the analysis 25 patients who were diagnosed with hypothyroidism at our hospital and received levothyroxine before TgAb and TPOAb were measured or thyroid ultrasound was performed. Finally, 237 patients with primary hypothyroidism were included in this study.

### Diagnostic procedures and tests for chronic thyroiditis

The diagnostic criteria for hypothyroidism were elevated TSH with normal or decreased FT4. Thyroid hormones (TSH, FT3, and FT4) were measured by the CLEIA assay using Lumipulse Presto II (FUJIREBIO Inc., Tokyo). Institutional reference values were used as follows: TSH 0.75–4.12 μIU/mL, FT3 2.51–3.47 pg/mL, FT4 0.68–1.26 ng/mL. Thyroid-related autoantibodies (TgAb, TPOAb, TSAb, TRAb) were measured by ECLIA method using cobas e 801 (Roche Diagnostics K.K, Switzerland). Positive results were considered when TgAb ≥ 16.0 IU/mL and TPOAb ≥ 28.0 IU/mL, TSAb ≥ 120%, and TRAb ≥ 2.0 IU/mL. Among the TRAb, immunoglobulin or antibodies that exhibit TSH-like stimulatory activity were measured as TSAb.

**Figure 2:**
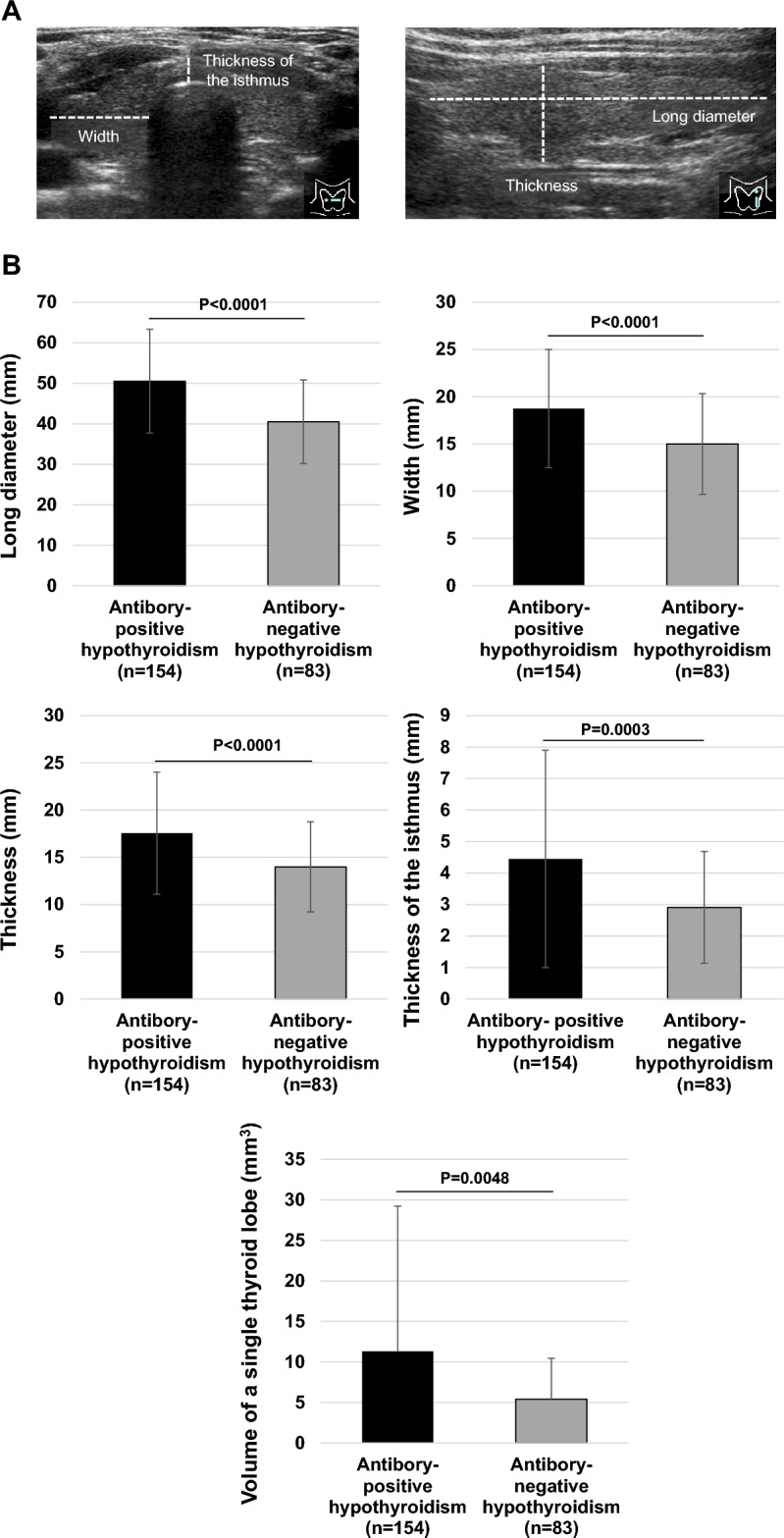
Correlation between various findings on thyroid ultrasonography in participants with seropositive and seronegative chronic thyroiditis. (**A**) Long diameter, width, thickness, and thickness of the isthmus were evaluated in this study. (**B**) The long diameter, width, thickness, isthmus thickness, and volume of a single thyroid lobe were evaluated in participants of seropositive and seronegative chronic thyroiditis.

In this study, we evaluated thyroid ultrasonographic findings that are characteristic of chronic thyroiditis, a frequent cause of primary hypothyroidism, based on the criteria of the Japan Thyroid Association (https://www.japanthyroid.jp/en/guidelines.html#Hyp)^4^. These findings are enlargement/atrophy, internal coarse, irregular margins. Thyroid ultrasonography was performed by at least two skilled sonographers with ultrasonographic equipment, Aplio series (CANON MEDICAL SYSTEMS, Tochigi, Japan). The site of thyroid measurement on ultrasonography is shown in Fig. 2. The long diameter, width, thickness, and isthmus thickness of the thyroid gland were measured with ultrasonography. We diagnosed an enlarged goiter when the thyroid gland met one of the following criteria: diameter greater than 50 mm, width greater than 20 mm, thickness greater than 15 mm, or isthmus thickness greater than 3 mm. Internal coarseness and margin irregularity were determined subjectively. We determined hypervascularity when the maximal velocity of blood flow in the superior thyroid artery was 39 cm/sec or greater. Thyroid volume was calculated using the following formula (Long diabeter*Thickness*Width*π/6) using data obtained from the larger left and right thyroid lobes^5^.

### Statistical analysis

Data are expressed as mean and standard deviation. The primary objective of this study was the positivity rate of thyroid-related autoantibodies (TgAb, TPOAb) in patients with primary hypothyroidism in our hospital. A secondary evaluation was to assess the structural differential position of chronic thyroiditis with and without antibodies. Mann–Whitney’s *U* test and chi-square test were used to evaluate the association between the clinical background of antibody-positive/negative hypothyroidism and thyroid ultrasound findings. To evaluate whether there is a difference in TgAb or TPOAb levels at the dose of levothyroxine that results in TSH level less than 10 μIU/mL, antibody-negative and positive patients were divided into three quartiles by each antibody level and evaluated using ANOVA. The Tukey test was used as the post hoc test. Nominal logistic analysis by age, sex, and TSH level was used to evaluate factors involved in positive/negative TgAb and TPOAb. Because the antibody and thyroid hormone values were non-normally distributed, they were natural logarithmized for analysis between groups and when calculating Spearman's rank correlation coefficient with levothyroxine dosage. When the antibody titer was lower than the assay's sensitivity, the original deal was replaced by 0.1 for analysis. Participants whose thyroid hormone levels were not within the measurement sensitivity were excluded when evaluating the correlation coefficient. For analysis, we used JMP (16.0.1) and EXCEL for Mac (16.58) for tabulation.

### Informed consent

Consent was obtained for participants in this study via opt-out on the Kawasaki Medical School website.

## Results

### Clinical characteristics of this study participants

The clinical characteristics of the patients are shown in Table 1. The overall positive rate of autoantibodies among the participants was 62.0% for TgAb, 49.4% for TPOAb. In participants with antibody-positive hypothyroidism, the positive rates of TgAb and TPOAb were 94.8% and 75.5%, respectively. Both TgAb and TPOAb were negative in 34.6% of all participants. Participants of antibody-negative hypothyroidism were older than those with participants of antibody-positive hypothyroidism (71.6 ± 12.8 years vs. 59.0 ± 16.4 years) and had a higher proportion of males (70.7% vs. 38.7%, p < 0.001). Factors related to positive/negative TgAb and TPOAb were younger age (p < 0.0001, p = 0.0019, respectively) and female sex (p = 0.0036, 0 = 0.0047, respectively). Furthermore, in a multiple logistic analysis, TSH level at the time of antibody measurement, was not an independent predictive factor for antibody positivity (TgAb: p = 0.82, TPOAb: p = 0.60). Among all participants, TRAb and TSAb were measured in 123 and 86 participants, respectively, with positive rates of 8.1% and 50.0%.

**Table 1:** Comparison of various parameters between antibody-positive and negative hypothyroidism.

Parameter	All participants (n = 237)	Antibody-positive hypothyroidism (n = 155)	Antibody-negative hypothyroidism (n = 82)	p value
Male/female	118/119	60/95	58/24	< 0.001
Age (years)	63.3 ± 16.4	59.0 ± 16.4	71.6 ± 12.8	< 0.001
Percentage of TgAb-positive patients (%)	62.0	94.8	0	< 0.001
Percentage of TPOAb-positive patients (%)	49.4	75.5	0	< 0.001
Percentage of TRAb-positive patients (%)	8.1	10.7	2.6	0.12
Percentage of TSAb-positive patients (%)	50.0	45.6	58.6	0.25

Data are presented as mean ± standard deviation.

*TgAb* anti-thyroglobulin antibody, *TPOAb* anti-thyroid peroxidase antibody, *TRAb* anti-TSH receptor antibodies, *TSAb* anti-TSH receptor stimulating antibody, *TSH* thyroid-stimulating hormone.

### Differences of thyroid-related autoantibodies and findings in thyroid ultrasonography

Next, we examined differences in thyroid morphologic changes assessed by thyroid ultrasonography in participants with antibody-positive or negative hypothyroidism. Thyroid size results on thyroid ultrasound are shown in Fig. 2B. Long diameter in antibody-positive or negative hypothyroidism was 50.5 ± 12.8 mm and 40.5 ± 10.3 mm, width was 18.7 ± 0.8 mm and 15.2 ± 1.2 mm, respectively. Thickness was 17.6 ± 6.5 mm and 14.0 ± 4.8 mm, respectively, and thickness of the isthmus was 4.5 ± 3.5 mm and 2.9 ± 1.8 mm, respectively (p < 0.005). Compared to antibody-negative hypothyroidism, antibody-positive hypothyroidism tended to have a larger thyroid gland size at diagnosis. Table 2 shows the results of thyroid ultrasonographic findings regarding the internal properties of the thyroid gland. Internal rough construction was significantly more common in seropositive chronic thyroiditis (p < 0.05). The prevalence of margin irregularity and increased blood flow did not differ between the two groups. Thyroid nodules and cysts were significantly more common in participants with antibody-negative hypothyroidism.

**Table 2:** Comparison of various parameters related to thyroid ultrasonography between participants of antibody-positive and negative hypothyroidism.

Parameter	Antibody-positive hypothyroidism (n = 155)	Antibody-negative hypothyroidism (n = 82)	p value
Thyroid gland enlargement (%)	63.6	36.6	< 0.0001
Internal rough construction (%)	69.5	54.9	0.026
Margin irregularity (%)	58.3	52.4	0.39
Increased blood flow (%)	59.6	53.1	0.34
Thyroid nodules (%)	38.2	51.2	0.074
Thyroid cysts (%)	15.8	43.9	< 0.0001

Data are presented as mean ± standard deviation.

### Thyroid hormones and thyroid-related autoantibodies

**Figure 3:**
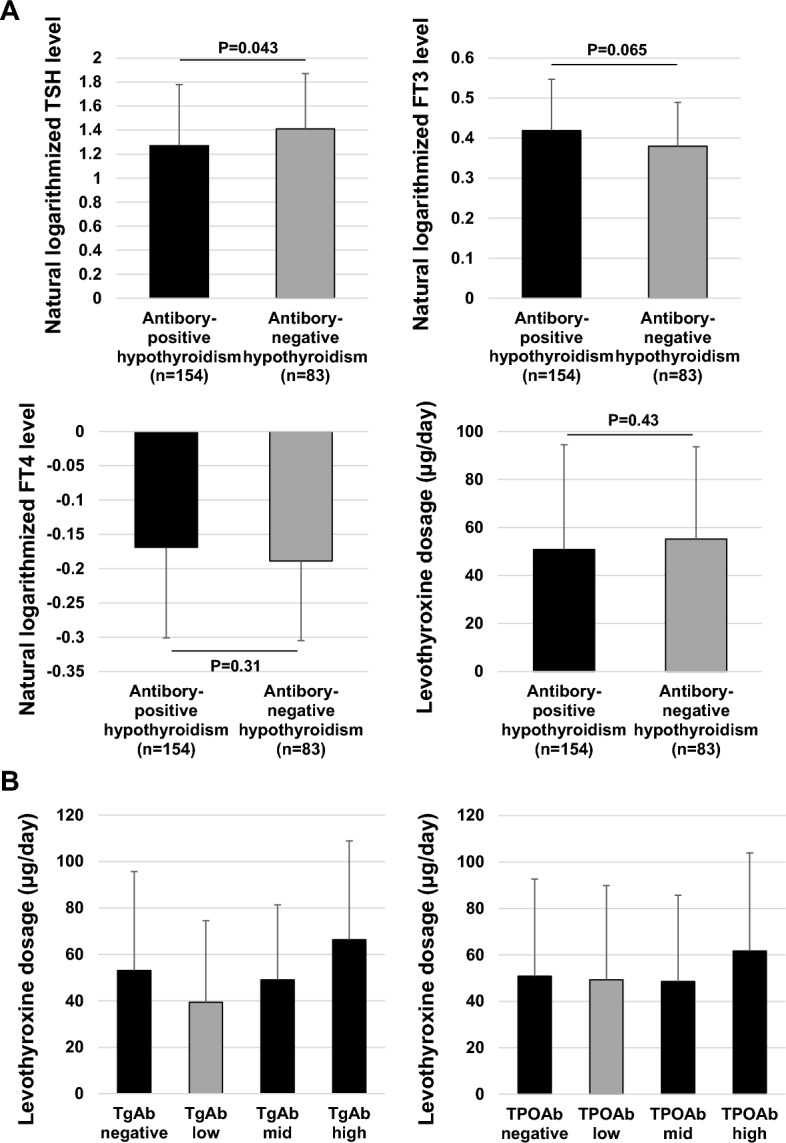
Thyroid hormone levels and levothyroxine dosage in participants with seropositive and seronegative chronic thyroiditis. (**A**) TSH, FT3, and FT4 at the initial visit and levothyroxine dosage at the time when hypothyroidism was improved in participants with seronegative and seropositive chronic thyroiditis. (**B**) Levothyroxine requirements to improve to 10 μIU/mL TSH were assessed for each TgAb and TPOAb level. Antibody-positive participants were divided into three quintiles according to antibody level.

Figure 3A shows the thyroid hormone levels at the initial visit in participants diagnosed with s antibody-positive or negative hypothyroidism. The mean TSH was 42.1 ± 70.8 and 46.3 ± 64.1, respectively, and was significantly higher in the seronegative participants (p = 0.043). There were no group differences in mean FT3 and mean FT4 levels. The levothyroxine dosage at the time of improvement of hypothyroidism was 50.8 ± 38.4 μg/day and 55.2 ± 43.6 μg/day, respectively, with no significant difference. The correlation between thyroid-related autoantibody levels and levothyroxine dosage in all participants in this study is shown in Fig. 3B. There was no difference in levothyroxine requirement between participants with negative TgAb and those with positive TgAb at any level (p = 0.21 vs. TgAb low, p = 0.94 vs. TgAb mid, p = 0.23 vs. TgAb high). On the other hand, levothyroxine requirements were significantly higher in participants with high TgAb compared to those with low TgAb (p = 0.0040). TPOAb antibody levels and levothyroxine requirements were not different in each group.

## Discussion

In this study, we determined the positivity rate of thyroid-related autoantibodies at the time of diagnosis of primary hypothyroidism. The positive rates of TgAb and TPOAb in patients with primary hypothyroidism attending our hospital were 62.0% and 49.4%, respectively. We also found differences in thyroid ultrasound findings and thyroid hormone levels at the initial visit between antibody-positive and negative patients with primary hypothyroidism. The data of this study may be clinically useful, especially in the diagnosis of antibody-negative hypothyroidism.

Most cases of spontaneous hypothyroidism show positive for TgAb and/or TPOAb^6^, and measurement of these antibodies is useful in the diagnosis of chronic thyroiditis. TPOAb correlates with the degree of lymphocytic infiltration of thyroid tissue and the rate of thyroid destruction^7,8^. In this study, TPOAb-positive cases had significantly more thyroid ultrasound findings of thyroid gland enlargement and internal rough construction, which may reflect tissue destruction. The positivity rates of TgAb and TPOAb in the participants of this study were as low as 62.0% and 49.4%, respectively; in cases where both or either TgAb and TPOAb were positive, the positive rates of TgAb and TPOAb were 94.6% and 72.3%, respectively. A review on thyroid autoantibodies reported positive rates of 90–95% for TPOAb and 30–60% for TgAb in patients with autoimmune thyroiditis^9^. In contrast, we found higher frequency of positive TgAb than TPOAb. TgAb is positive in 10–15% of patients with non-autoimmune thyroid disease^10^. Most recent reports on TgAb positivity have reported associations with differentiated thyroid cancer^11^, and few reports have focused exclusively on patients with recent primary hypothyroidism. Differences in TgAb assays in patients with differentiated thyroid cancer may lead to misclassification of results^12^. The different trends in the positivity rates of TgAb and TPOAb in hypothyroid patients between this study and previous reports may be due to differences in the proportion of non-immune hypothyroidism included, patient backgrounds, and assay methods.

This study suggests that being male and older age may be independent factors for antibody-negative hypothyroidism. Therefore, antibody positivity may vary depending on the characteristics of the study participants and should be reported in different generations, races, and geographic areas.

A previous study of patients with primary hypothyroidism reported that 20.8% of chronic thyroiditis patients were both TgAb and TPOAb negative^13^. Patients with chronic thyroiditis who are both negative for TgAb and TPOAb have a higher FT4 and lower TSH with a more moderate clinical picture than antibody-positive cases^14^. In this study, 34.6% of participants were antibody-negative hypothyroidism. Clinically, however, participants with antibody-negative hypothyroidism had higher TSH and lower FT4 compared to antibody-positive participants. In addition, the participants of antibody-negative hypothyroidism in this study were more likely to be male and older than the participants of antibody-positive hypothyroidism. The previous reports were based on participants with younger age and there was no difference in terms of gender or age^14^. Few reports have included elder participants as in this study. Elderly male patients with hypothyroidism may be characterized by seronegativity.

TSAb is a useful marker for the diagnosis of Graves' disease^15,16^. Regarding the positivity rate of TSAb in chronic thyroiditis, it has been reported that TSAb is positive in 33% of TRAb-positive cases. On the other hand, in the present study, the positive rate of TSAb was 50.0% while that of TRAb was 8.1%. The positive rate of TRAb in chronic thyroiditis is around 0–48% and positively correlates with pretreatment TSH^15^. Further studies are needed about the positivity rate of TSAb in hypothyroidism.

There are several limitations to this study. First, this study is a single-center, retrospective, observational study. Among the patients with hypothyroidism in our hospital, 286 patients did not have thyroid-related autoantibodies measured or thyroid ultrasound. This could have a significant impact on the results. Second, the diagnostic criteria for chronic thyroiditis in this study was based on the diagnostic criteria proposed by the Japanese Thyroid Society. However, the diagnosis of clinically seronegative chronic thyroiditis was made using an adjunctive ultrasound examination when the TgAb and TPOAb results were negative. On the other hand, a reliable diagnosis of seronegative chronic thyroiditis requires evaluation of lymphocytic infiltration of the thyroid gland by needle biopsy. At our institution, we perform needle biopsy when ultrasonography is suspicious for malignant thyroid disease, but needle biopsy is rarely performed in the absence of obvious malignant findings. Indeed, most of the participants in this study did not have a pathology diagnosis. In addition, it was not possible to assess retrospectively from medical records regarding iodine intake. Therefore, this study does not rigorously differentiate between diseases such as malignant thyroid disease and excess/deficient iodine intake. Lastly, although the study evaluated the prevalence of TRAb and TSAb antibody positivity, there is a possibility that some bias may have occurred due to the small number of cases in this study.

In conclusion, the present study reveals a positive rate of thyroid-associated autoantibodies at the time of diagnosis of primary hypothyroidism. The study also revealed structural differences on thyroid ultrasound in antibody-positive/negative hypothyroid patients. The results of this study may be particularly useful in the diagnosis of antibody-negative hypothyroidism. Further studies are needed to determine the long-term clinical course of antibody-positive and antibody-negative patients.

## Data Availability

All data generated or analysed during this study are included in this published article.
